# Supplementary material for: Metamorphosis Imposes Variable Constraints on Genome Expansion through Effects on Development
Source: Integr Org Biol. 2023 Apr 18;5(1):obad015. doi: 10.1093/iob/obad015 (PMC10153748; doi:10.1093/iob/obad015)
Supplement: obad015_Supplemental_Files [file obad015_supplemental_files.zip › genomeSize_analyses.pdf]

Phylogenetic Analysis of Salamander Genome Size

Clay Cressler

2/16/2023

This markdown script contains all of the code necessary to replicate the phylogenetic comparative analyses of salamander genome size found in the manuscript, “Mueller et al. Metamorphosis imposes variable constraints on genome expansion.”

I will proceed with fitting the following models: a standard Brownian motion model (BM1), a Brownian motion model where the drift parameter (σ) varies across regimes (BMS), an Ornstein-Uhlenbeck (OU) model where only θ varies across regimes (OUM), an OU model where selection strength varies across regimes (α) (OUMA), an OU model where drift varies across regimes (OUMV), and an OU model where both selection and drift vary across regimes (OUMVA).

It is clear from the table below summarizing the model fits that the multiple-α models (OUMA and OUMVA) produce fitting errors. This can be seen in the log-likelihoods, which if believed are often several orders of magnitude better than simpler models, and from the parameter estimates, which often include at least one parameter that is estimated to be approximately 0. The only model where neither of these seeming errors was OUMA for the simplest adaptive hypothesis (meta.other), which is perhaps unsurprising because that was one of the only models where OUwie did not warn about not having enough data to fit the model. Because almost all of the multiple-α models produced fitting errors, we have decided to discard all of them and focus only Brownian motion models (BM1 and BMS); single-α, single-σ models (OUM); and single-α, multiple-σ models (OUMV).

```
##                       Hypothesis  loglik npars    AICc                    alpha
## 1   abrupt.gradual.dd.paed.OUMA 4360.00     9 -8710.0 0.000605, 53.7, 100, 92.9
## 2              meta.dd.paed.OUMA 3890.00     7 -7760.0          37.1, 100, 87.4
## 3     abrupt.gradual.other.OUMA 2820.00     7 -5620.0        38, 9.81, 3.86e-05
## 4    abrupt.gradual.other.OUMVA 2220.00     9 -4420.0          42.7, 39.2, 17.9
## 5  abrupt.gradual.dd.paed.OUMVA 1850.00    12 -3680.0    2.54, 42.1, 43.9, 1.02
## 6                meta.other.OUMA   -7.87     5    26.3               0.239, 1.21
## 8               meta.other.OUMVA   -7.51     6    27.8            1.01e-09, 1.38
## 10            meta.dd.paed.OUMVA   -5.04     9    29.7     1.94, 1.09e-09, 0.348
##                            sigma                          theta
## 1                      3.16e-05 2.21e-36, 0, 2.44e-16, 3.28
## 2                        3.2e-05    2.13e-28, 2.77, 1.32e-26
## 3                      0.000125    3.28, 1.18e-08, 5.11e-15
## 4   0.642, 3.18e-05, 3.37e-05           3.7, -0.761, -17.7
## 5  2.13, 1.57, 3.46e-05, 6.73    0.0104, 3.39, 126, -1490
## 6                         0.455                  3.62, 4.19
## 8                  0.459, 0.356                  3.67, 4.12
## 10        0.266, 0.455, 0.281            3.75, 3.58, 5.64
```

Of these, the best-fitting model is the OU model with 4 selective regimes, with separate drift parameters for each regime. Here are the negative log-likelihoods, number of parameters, AICc values, and parameter estimates for each of the models.

```
##                    Hypothesis loglik npars AICc alpha
## 7  abrupt.gradual.dd.paed.OUMV  -3.43     9  0.0  1.29
## 9    abrupt.gradual.other.OUMV  -6.94     7  2.4  1.64
## 11           meta.dd.paed.OUMV  -7.40     7  3.3 0.946
## 12             meta.other.OUMV -10.30     5  4.5  1.22
## 13            meta.dd.paed.BMS -11.90     4  5.6    NA
## 14    abrupt.gradual.other.OUM -11.60     5  7.3  2.07
## 15  abrupt.gradual.dd.paed.OUM -10.70     6  7.6  2.46
## 16  abrupt.gradual.dd.paed.BMS -11.90     5  7.7    NA
## 17              meta.other.OUM -13.00     4  7.8  1.86
## 18              meta.other.BMS -14.10     3  7.9    NA
## 19            meta.dd.paed.OUM -12.50     5  9.1  2.14
## 20    abrupt.gradual.other.BMS -13.90     4  9.6    NA
## 21                         BM1 -19.10     2 15.8    NA
##                          sigma                                       theta
## 7    0.842, 0.374, 0.6, 0.364                         3.73, 2.69, 3.66, 4.3
## 9         0.389, 0.634, 0.835                              2.98, 3.64, 4.05
## 11         0.826, 0.521, 0.37                               4.3, 3.55, 4.62
## 12               0.547, 0.81                                    3.56, 4.37
## 13        0.783, 0.474, 0.349          3.82, 5.52e-12, 2.36e-10, 1.41e-10
## 14                      0.792                              3.01, 3.63, 4.04
## 15                      0.812                        3.76, 2.99, 3.62, 4.18
## 16 0.783, 0.447, 0.484, 0.359 3.82, 5.78e-12, 1.1e-11, 2.27e-10, 1.38e-10
## 17                      0.786                                    3.53, 4.18
## 18               0.458, 0.759                        3.7, 3.14e-10, 5.65e-11
## 19                      0.803                              3.93, 3.51, 4.26
## 20        0.399, 0.485, 0.762          3.69, 2.65e-11, 2.85e-10, 5.82e-11
## 21                      0.683                                3.77, 3.77e-10
```

One way to help visualize what this best-fitting model is telling us is by looking at the distribution of genome sizes for species in each selective regime, relative to the expected stationary distribution of genome sizes, given the model estimates for
θ,
α, and
σ. In particular, Ho and Ane (2013) showed that the stationary distribution of an OU process will be a normal distribution with mean
θ and variance
$\sigma^2/(2\alpha)$. From this figure, it is clear that genome size is under strong stabilizing selection in the direct development regime (which is by far the most common selective regime across the tree). This also appears to be true of genome size in salamander that undergo gradual metamorphosis (although since the peak of the genome size distribution is slightly smaller than the predicted mean, there are still some salamanders potentially experiencing some directional selection towards increasing genome sizes). Genome size in salamanders that undero abrupt metamorphosis, on the other hand, appears to be under directional selection towards smaller genome sizes, since almost all the

salamanders in this regime have genome sizes that are larger than the expected mean. Conversely, genome size in the paedomorph regime appears to be under directional selection towards larger size (which we know is not actually directional selection so much as unconstrained TE accumulation).

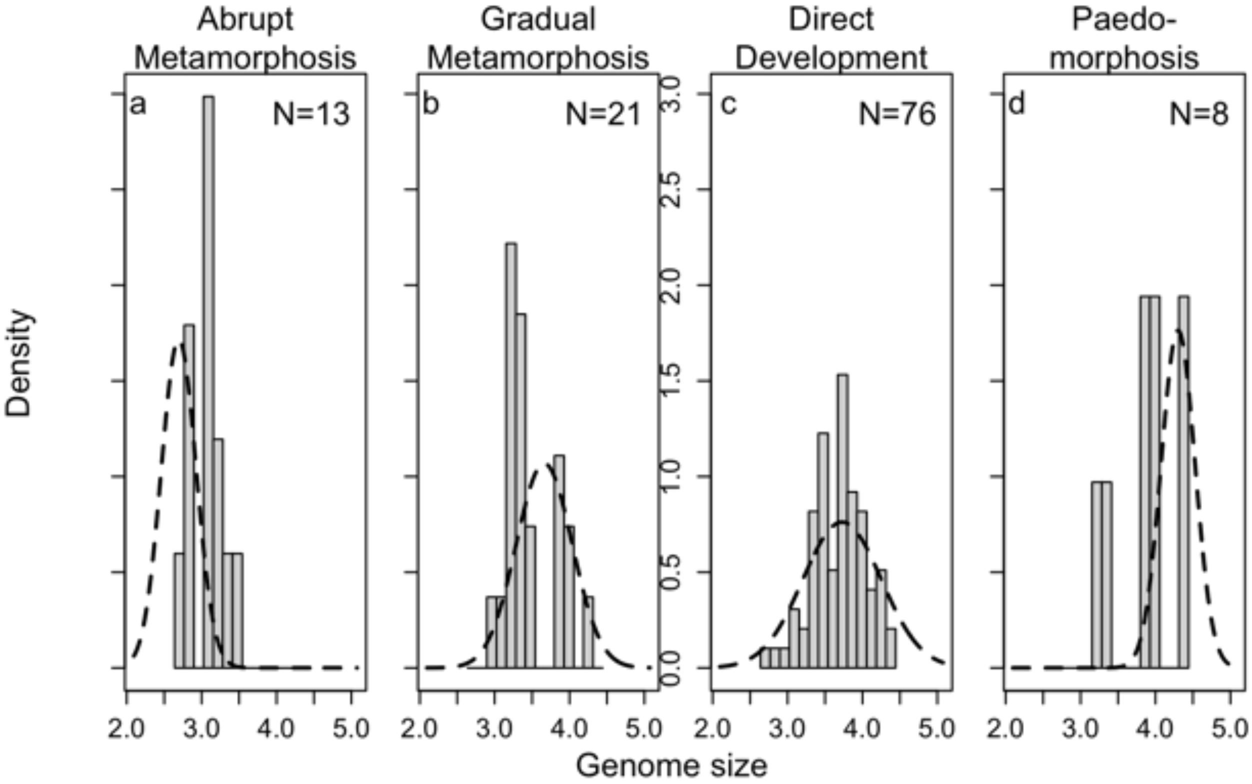

Figure 1: Observed distributions of genome size in each selective regime compared against the stationary distribution predicted by the OUMV model (dashed line).

Another way to think about this is to compute the average deterministic pull $\left(\sum_i \alpha_r(\bar{\theta}_r - X_{i,r})\right)/N_{i,r}$, where $X_{i,r}$ is the genome size of species i in regime r, $N_{i,r}$ is the total number of species in regime r and α_r is the magnitude of the deterministic pull towards the expected genome size θ_r, and to compare that to the magnitude of the stochastic drift in genome size for species in that regime, σ_r. From this, we see that there is no tendency for genome size evolution in direct developers; salamanders who undergo abrupt metamorphosis appear to be experiencing a strong deterministic trend towards smaller genome sizes; interestingly, paedomorphic salamanders and salamanders who under gradual metamorphosis are both experiencing a determinstic trend towards larger genome sizes that is stronger than any stochastic tendency towards drift. (**Note**: I do worry that this is overinterpreting the parameter estimates.)

```
##                     Deterministic pull Stochastic drift
## direct development           0.0696013        0.8421015
## meta-abrupt                 -0.4798658        0.3739580
## meta-gradual                 0.2592405        0.6003767
## paedomorphosis               0.5293197        0.3637319
```

However, it is important to assess how strong the support for this hypothesis truly is. Following the protocol of Boettiger et al. (2012) for phylogenetic Monte Carlo, I need to simulate datasets under different models and ask how often the true (data-generating) model is rejected in favor of an alternative model.

We will do the following pairwise comparisons of models:

1. $metamorphosis - other$ (OUMV) versus BM.
2. $meta - paed - dd$ (OUMV) versus $metamorphosis - other$ (OUMV).
3. $meta_{abrupt} - meta_{gradual} - other$ (OUMV) versus $metamorphosis - other$ (OUMV).
4. $meta_{abrupt} - meta_{gradual} - paed - dd$ (OUMV) versus $meta - paed - dd$ (OUMV).
5. $meta_{abrupt} - meta_{gradual} - paed - dd$ (OUMV) versus $meta_{abrupt} - meta_{gradual} - other$ (OUMV).
6. $meta_{abrupt} - meta_{gradual} - paed - dd$ (OUMV) versus $meta_{abrupt} - meta_{gradual} - paed - dd$ (OUM)

Comparison (1) tests whether we can confidently reject a non-adaptive hypothesis for genome size evolution. Comparisons (2) and (5) test whether distinguishing direct development and paedomorphosis is supported. Comparisons (3) and (4) test whether distinguishing gradual and abrupt metamorphosis is supported. Comparison (6) tests whether allowing separate drift parameters for each regime is supported. We will simulate datasets under all of the models above, and then fit data generated by each of the two models under comparison to each of those models.

We can also use these fits to look at the confidence intervals for each of the parameters of the best-fitting model using a parametric bootstrap.

```
##                  parameter Estimate           CI
## 1                    alpha    1.290  0.403, 3.93
## 2            sigma.sq (dd)    0.842   0.73, 1.08
## 3  sigma.sq (meta-abrupt)    0.374 0.214, 0.521
## 4 sigma.sq (meta-gradual)    0.600 0.399, 0.895
## 5          sigma.sq (paed)    0.364 0.136, 0.629
## 6               theta (dd)    3.730    2.93,4.63
## 7     theta (meta-abrupt)    2.690    1.07,3.44
## 8    theta (meta-gradual)    3.660    3.39,3.93
## 9             theta (paed)    4.300    3.87,5.19
```

To determine whether the data really supports the conclusions we want to draw from the AICc table, we use the phylogenetic Monte Carlo approach of Boettiger et al. 2012. We define the difference in the log-likelihoods between the two models as our test statistic: $\delta = -2(\log L_0 - \log L_1)$, where we assume that the L_1 is the likelihood of the more complex model. Higher values of this likelihood ratio indicate more support for the complex model. We will use the simulated datasets to estimate the distribution of δ when the data is generated by either of the two models under comparison. Our first comparison is between the $meta - other$ hypothesis, fitted with the OUMV model (the simplest adaptive hypothesis, but with separate σ parameters for each regime), and the non-adaptive Brownian motion hypothesis. The p-value for this comparison is 0.004: when the data is generated by a non-adaptive model, the value of the test statistic is almost never as large as the value that we observed with the true dataset.

```
## [1] 0
```

We can also look at the overlap between the distributions of the test statistic when the data is generated by the non-adaptive model versus the adaptive model to get a sense of power. You can see that the power is very high (0.94), meaning that 94% of the test statistic values when the data is generated by the adaptive model are larger than the 95th percentile of the distribution when the data is generated by the non-adaptive model. Both the p-value and the power can be visualized by a plot of the two distributions (Fig. 2A).)

```
## [1] 0.968
```

Using the same methodology, we can calculate the approximate p-value and power for the second test, between $meta - paed - dd$ (OUMV) and $metamorphosis - other$ (OUMV). This tests whether more finely subdividing non-metamorphosing strategies into direct development and paedomorphosis provides a better fit to the data than simply between distinguishing between metamorphosing and non-metamorphosing strategies. Here the p-value is 0.088 and the power is 0.57, suggesting that the addition of direct development and paedomorphosis as separate constraint regimes does not really improve the explanatory power of the model. See Fig. 2B for a visual depiction of these results.

```
## [1] 0.118
```

```
## [1] 0.564
```

Using the same methodology, we can calculate the approximate p-value and power for the third test, $meta_{abrupt} - meta_{gradual} - other$ (OUMV) and $metamorphosis - other$ (OUM). This tests whether distinguishing between different metamorphosis strategies is supported. Here the p-value is 0.084 and the power is 0.522. See Fig. 2C for a visual depiction of these results.

```
## [1] 0.066
```

```
## [1] 0.622
```

We also calculate the p-value and power for the fourth test, $meta_{abrupt} - meta_{gradual} - paed - dd$ (OUMV) versus $meta - paed - dd$ (OUMV). This tests whether separate regimes for abrupt and gradual metamorphosis are supported, after accounting for the two non-metamorphosing strategies (direct development and paedomorphosis). The more complicated model is strongly supported, with a p-value of 0.046 and power of 0.718. See Fig. 2D for a visual depiction of these results.

```
## [1] 0.04
```

```
## [1] 0.704
```

We also calculate the p-value and power for the fifth test, $meta_{abrupt} - meta_{gradual} - paed - dd$ (OUMV) versus $meta_{abrupt} - meta_{gradual} - other$ (OUMV). This tests whether allowing separate regimes for direct developers and paedomorphs is supported, once you have accounted for abrupt and gradual metamorphosis. For this comparison,

the p-value is 0.054 and the power is 0.728, indicating that there is fairly strong support for the more complex model. See Fig. 2E for a visual depiction of these results.

```
## [1] 0.064
```

```
## [1] 0.684
```

Finally, just to cross all the t's and dot all the i's, we can calculate the p-value and power for the sixth test, $meta_{abrupt} - meta_{gradual} - paed - dd$ (OUMV) versus $meta_{abrupt} - meta_{gradual} - paed - dd$ (OUM), which tests whether multiple σ values is well-supported by the data. Here the p-value is 0.002 and the power is 0.96, suggesting very strong support for the more complex model. See Fig. 2F for a visual depiction of these results.

```
## [1] 0.006
```

```
## [1] 0.984
```

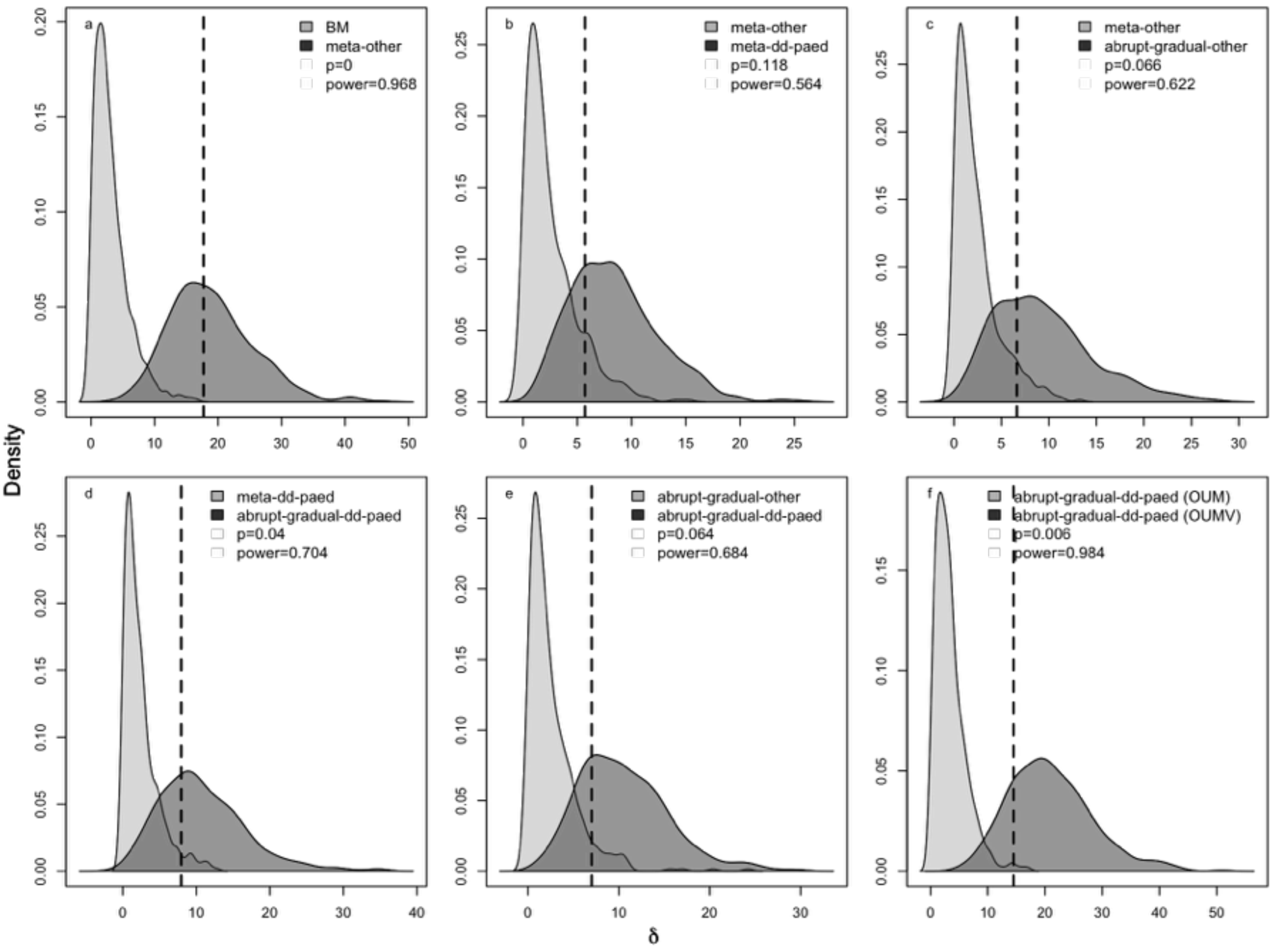

Figure 2: Comparing the distributions of the test statistic when the data is generated by simpler (light gray) or more complex (dark gray) evolutionary models. The dashed line gives the actual value of the test statistic for the true data.

Analyses with a different evolutionary history for plethodontids

In the above analyses, the ancestor of all plethodontids is assumed to be a metamorphosing amphibian. However, there is some debate about this, and some researchers think that this ancestor was actually a direct developer. As such, we want to make sure that the conlusions drawn above are unaffected by this assumption. This requires modifying all of the adaptive hypotheses to "repaint" the ancestral nodes that are currently coded as metamorphosis.

As with the original evolutionary history, the OU models with variable α produced nonsensical fitting results.

```
##                      Hypothesis  loglik    AICc                         alpha
## 1             meta.other.OUMVA 4720.00 -9420.0               100, 7.63e-05
## 2              meta.dd.paed.OUMA 3460.00 -6910.0          18.2, 100, 6.06e-09
## 3             meta.dd.paed.OUMVA 3470.00 -6910.0             18.2, 99.9, 37.5
## 4                meta.other.OUMA 3450.00 -6890.0                     99.9, 18
## 5   abrupt.gradual.dd.paed.OUMA   -3.36    26.4      2.39, 1.02, 0.74, 0.641
## 7     abrupt.gradual.other.OUMA   -6.94    28.9           0.609, 0.428, 1.91
## 8    abrupt.gradual.other.OUMVA   -5.24    30.1           3.89, 0.178, 2.21
## 10 abrupt.gradual.dd.paed.OUMVA   -1.65    30.3 3.72, 5.43e-06, 0.777, 1.09
##                            sigma                      theta
## 1           6.95e-05, 3.66e-05             3.28, 1.37e-26
## 2                        3.2e-05  2.48e-14, 3.17, -1.11e-34
## 3  3.61e-05, 3.2e-05, 3.16e-05  -5.07e-20, 3.27, 3.44e-12
## 4                       3.17e-05             3.24, 7.94e-19
## 5                          0.491      3.61, 1.69, 3.68, 4.7
## 7                          0.499           1.84, 3.69, 3.95
## 8          0.0691, 0.503, 0.414           3.28, 3.72, 3.95
## 10   0.324, 1.36, 0.553, 0.334 3.63, -1290000, 3.68, 4.34
```

Excluding these poor-fitting models, the top model remains unchanged from the previous analysis: $meta_{abrupt} - meta_{gradual} - dd - paed$ is still the best-fitting hypothesis. There is some reordering of the other models, however, in that the multiple-σ stochastic models (BMS) outperform some of the multiple-σ adaptive models. However, none of these stochastic, Brownian motion models outperform their adaptive, OU models (e.g., every OUMV model outperforms the corresponding BMS model). Thus, our overall evolutionary conclusions remain unchanged by modifying the selective regime history of plethodontid salamanders.

```
##                    Hypothesis loglik AICc alpha                       sigma
## 6  abrupt.gradual.dd.paed.OUMV  -4.16 28.0  1.35 0.842, 0.382, 0.608, 0.375
## 9            meta.dd.paed.OUMV  -7.61 30.2 0.888         0.83, 0.507, 0.354
## 11            meta.dd.paed.BMS -11.00 30.4    NA        0.791, 0.458, 0.299
## 12   abrupt.gradual.other.OUMV  -8.00 31.0  1.59        0.388, 0.632, 0.836
## 13  abrupt.gradual.dd.paed.BMS -10.60 31.7    NA 0.796, 0.376, 0.491, 0.298
## 14             meta.other.OUMV -10.80 32.1  1.16               0.534, 0.814
## 15  abrupt.gradual.dd.paed.OUM -10.70 34.2  2.48                      0.814
## 16              meta.other.BMS -14.00 34.3    NA               0.453, 0.758
## 17    abrupt.gradual.other.BMS -13.60 35.6    NA        0.375, 0.484, 0.761
## 18    abrupt.gradual.other.OUM -12.70 36.0  1.95                      0.791
## 19              meta.other.OUM -13.90 36.2  1.75                      0.784
## 20            meta.dd.paed.OUM -13.00 36.5  2.14                      0.806
## 21                         BM1 -19.10 42.3    NA                      0.683
##                                           theta
## 6                        3.54, 2.55, 3.66, 4.29
## 9                               4.2, 3.59, 4.6
## 11           3.86, 4.6e-12, 2.16e-10, 1.65e-10
## 12                               2.94, 3.64, 4
## 13 3.86, 4.15e-12, 1.83e-12, 2.06e-10, 1.74e-10
## 14                                   3.59, 4.31
## 15                       3.62, 2.84, 3.62, 4.18
## 16                      3.73, 3.1e-10, 6.36e-11
## 17          3.73, 6.49e-12, 2.99e-10, 6.79e-11
## 18                             2.93, 3.63, 3.95
## 19                                   3.55, 4.11
## 20                             3.81, 3.52, 4.26
## 21                              3.77, 3.77e-10
```

Analyses with a different handling of the root state

OUwie has different options for dealing with the root state, $X(0)$. Absent any information about the phenotype deep in the tree, this parameter is often very difficult to estimate in OU models (Cressler, et al. 2015). One alternative is to assume that the value of $X(0)$ is distributed according to the stationary distribution of an OU process, which eliminates this parameter by absorbing the variance into the covariance matrix implied by the phylogeny itself (Ho and Ané 2013). However, OUwie does not currently support this approach for OU models with multiple α or σ parameters, and as far as we know, the mathematical modifications for these models have not been worked out. Because the option to estimate the value of the root node as in the earlier implementations (Hansen 1997; Butler and King 2004; and specified in OUwie by setting root.station=FALSE) is available for all of the models of interest, we used it for all of the model fitting above. Here we explore the implications of that decision for the parameter estimates and model fits. We can only do this, of course, for the single-α, single-σ models.

The AICc estimates are not dramatically affected by handling of the root state: the order of the best-fitting models is the same in either case. (Note, however, that the best model in the single-α, single-σ case is not the same as the best model when σ is allowed to vary amongst the regimes, and that the AICc differences in model fits are smaller, suggesting that the support for the hypotheses is weaker.)

```
##                          Estimating.root Stationary.root
## abrupt-gradual-other               33.81           34.02
## abrupt-gradual-dd-paed             34.13           34.24
## meta-other                         34.29           34.57
## meta-dd-paed                       35.63           35.82
```

We also see almost no difference in the estimates of the θ values for each selective regime.

```
##                                 Estimating.root     Stationary.root
## meta-other                     3.01, 3.63, 4.04    3.02, 3.62, 4.03
## meta-dd-paed           3.76, 2.99, 3.62, 4.18 3.76, 3, 3.62, 4.18
## abrupt-gradual-other                 3.53, 4.18          3.53, 4.16
## abrupt-gradual-dd-paed       3.93, 3.51, 4.26    3.92, 3.51, 4.25
```

We see similar estimates of the strength of the deterministic pull, α, although the differences are somewhat larger.

```
##                          Estimating.root Stationary.root
## meta-other                          2.07            2.14
## meta-dd-paed                        2.46            2.52
## abrupt-gradual-other                1.86            1.94
## abrupt-gradual-dd-paed              2.14            2.22
```

Finally, the estimates of noise intensity σ are almost identical.

```
##                          Estimating.root Stationary.root
## meta-other                          0.63            0.64
## meta-dd-paed                        0.66            0.67
## abrupt-gradual-other                0.62            0.63
## abrupt-gradual-dd-paed              0.64            0.65
```

This suggests that the handling of the root state does not have a large effect on the parameter estimates or model fits.
